# Argatroban Treatment and Decreased Fibrinogen in a Septic Patient

**DOI:** 10.7759/cureus.7573

**Published:** 2020-04-07

**Authors:** Aman N Ajmeri, Amro Al-Astal, Shantanu Singh

**Affiliations:** 1 Internal Medicine, Joan C. Edwards School of Medicine, Marshall University, Huntington, USA; 2 Internal Medicine/Pulmonology, Joan C. Edwards School of Medicine, Marshall University, Huntington, USA

**Keywords:** argatroban, fibrinogen, disseminated intravascular coagulopathy (dic), sepsis, infectious endocarditis

## Abstract

Disseminated intravascular coagulation (DIC) is a consumptive coagulopathy associated with multiple conditions. Diagnosis is based upon clinical and laboratory findings with assessment of fibrinogen, platelets, D-dimer, prothrombin time/international normalized ratio and activated partial thromboplastin time. Herein, we report a case of a 39-year-old female patient diagnosed with endocarditis complicated by pulmonary septic emboli. For anticoagulation, the patient initially was treated with a heparin drip, but the patient remained subtherapeutic despite increasing dosage. The patient was transitioned to argatroban and developed an acute drop in the fibrinogen level. With concern for possible DIC, argatroban was held with a repeat panel six hours later revealing a significantly improved fibrinogen level. It was discovered that the Clauss method, which measures the capability of fibrinogen to form a clot after a high concentration of thrombin is added to diluted plasma, was used to measure fibrinogen at our institute. Argatroban may falsely reduce measured fibrinogen levels in vitro, caused by this method.

## Introduction

Disseminated intravascular coagulation (DIC) is a devastating syndrome that may complicate multiple medical and surgical conditions, including sepsis. It is described as a state associated with rapid systemic activation and consumption of platelet and coagulant factors leading to concurrent intravascular thrombosis and bleeding [1]. Abnormal laboratory findings include a prolonged activated partial thromboplastin time (aPTT) and prothrombin time (PT)/international normalized ratio, decrease in platelet count and fibrinogen, and elevated D-dimer [2]. Herein, we present a case of a patient with sepsis secondary to endocarditis complicated by septic pulmonary emboli, who received adequate antimicrobial coverage with anticoagulation and had an acute drop in fibrinogen levels on day 7 of therapy.

## Case presentation

A 39-year-old female presented to the emergency department with complaints of shortness of breath. She had a significant history of intravenous (IV) drug abuse and stated she had last used heroin one day prior. She had also been hospitalized at an outside facility a month prior for sepsis secondary to methicillin-resistant Staphylococcus aureus (MRSA) bacteremia and tricuspid valve endocarditis. At that time she was treated with IV antibiotics and discharged on linezolid, but she did not complete the full treatment course. The patient had also been diagnosed with a pulmonary embolism during that hospitalization and was discharged on apixaban. She had taken her last dose of apixaban the day before presenting to the ER for this admission. Review of systems was significant for pleuritic chest pain, a cough with no sputum production that also exacerbated chest pain, nausea and vomiting for the past week.

Initial vitals were as follows: temperature 102°F, pulse rate 145 beats per minute, respiratory rate of 35 breaths per minute and oxygen saturation of 98% on room air. Physical exam revealed track marks that were present on the left neck. Chest auscultation revealed that the patient was tachycardic with an early systolic murmur at the left sternal border and shallow but clear breath sounds bilaterally. Old scarring from an abscess on the right thigh and track marks were present behind the right knee.

Initial workup revealed an elevated lactic acid of 3.1 mmol/L, leukocytosis with white blood cell count of 22.4 k/cmm, elevated D-dimer, an elevated B-type natriuretic peptide (BNP) 2,229 pg/ml, a mildly elevated troponin of 0.093 ng/ml that trended to 0.085 ng/ml and finally 0.054 ng/ml. Cultures were drawn, and the patient was started on vancomycin and piperacillin/tazobactam. A transthoracic echo revealed a 1.3-cm vegetation on the posterior leaflet of the tricuspid valve confirmed with a transesophageal echocardiogram. The CT revealed multiple occlusive emboli in her pulmonary artery involving the right lobar segment and segmental branches of lower limbs bilaterally, along with extensive airspace disease with cavitations that were possibly septic. Blood cultures grew *Serratia, *and the patient’s antibiotics were optimized to piperacillin/tazobactam, rifampin, and linezolid due to concerns over possible non-clearance of previous MRSA infection.

The patient was initially started on a heparin drip while cardiothoracic surgery planned to operate on her to remove the vegetation. The patient’s white count steadily trended down and vitals became more stable. Unfortunately, the heparin Xa unfractionated level was low (initially 0.16 IU/ml), indicating subtherapeutic anticoagulation. On day 1, the patient received a total of 6,021 IU of heparin at an average infusion rate of 18 IU/kg/hr. On day 2, the patient received a total of 47,632.8 IU of heparin at an average infusion rate of 23 IU/kg/hr. On day 3, the patient received a total of 60,143.1 IU of heparin at an average dose rate of 31 IU/kg/hr. On day 4, the patient received 66,900 IU of heparin at an average dose rate of 35 IU/kg/hr. And on day 5, the patient received 71,716.8 IU at an average dose rate of 38 IU/kg/hr. The patient received 40 IU/kg bolus of heparin per hospital standards with every infusion change (approximately every six hours). Despite the increasing titration of heparin, the highest anti-Xa level was no more than 0.27 IU/ml. On the sixth day of admission, the patient started complaining of increasing right-sided chest pain and CT chest showed a worsening area of probable infarct in the right lung. With concern for increasing lung infarction in the setting of subtherapeutic anticoagulation, heparin was stopped and subsequently the patient was started on argatroban. The following morning (day 7), the fibrinogen level dropped significantly to 50 from 650 mg/dl. There was a significant concern for DIC, but clinically the patient showed no signs of active bleeding or clinical worsening.

In the setting of sepsis from an uncontrolled source (infective endocarditis), a sudden drop in fibrinogen levels alerted all providers to a possible coagulopathic state of DIC. Of note, the patient had no clinical signs of DIC and the platelet levels were regularly above the 400,000 k/cmm. In the interest of precaution, the patient had argatroban therapy held. Six hours later, the DIC panel showed a fibrinogen of 470 mg/dl and platelets remained above 400,000 k/cmm. Low molecular weight heparin was started. Four-hour post-administration Xa level was not therapeutic at 0.23 IU/ml. For that reason, low molecular weight heparin was stopped, and the patient was started on bivalirudin. The patient is currently scheduled for surgical resection of the vegetation by cardiothoracic surgery.

## Discussion

A rapid drop in fibrinogen can alert clinicians to a consumptive process and in the setting of endocarditis and sepsis, acute DIC is a reasonable concern. The diagnosis of DIC is both clinical and lab based, and with a mortality ranging from 40% to 80%; it can be a very alarming diagnosis [3]. Thrombocytopenia, low fibrinogen, and elevated D-dimer are sensitive, but not specific. Treatment is usually targeted at the underlying cause. 

Argatroban is a parenteral direct thrombin inhibitor that has strong recommendations to be used as an alternative choice for anticoagulation in patients with heparin-induced thrombocytopenia [4]. Supervising its anticoagulant activity is regularly achieved by the aPTT. The therapeutic range of argatroban is achieved by administering it to maintain a steady-state aPTT of 1.5-3.0 times the baseline value and without exceeding 100 seconds. Its anticoagulant properties occur via binding onto the active site on thrombin, leading to inhibition of thrombin-stimulated reactions, inclusive of fibrin formation, activation of coagulation factors V, VII, and XII, and platelet aggregation [5]. This can lead to clotting time prolongation. Of note, coagulation tests (aPTT, PT, and fibrinogen) are based on the measurements of clotting time [4].

Fibrinogen is a commonly used marker to investigate a coagulation state or for diagnosis of hemorrhagic disorders, with levels less than 1 g/l having a low sensitivity (22%) and high specificity (100%) for DIC [6]. It can be measured by a few different methods, including the Clauss method and immunological fibrinogen assay, amongst others. The Clauss method is one of the most commonly used techniques. It is done by adding high concentrations of thrombin to diluted plasma. Ross et al. evaluated the effects of direct thrombin inhibitors on the fibrinogen measurements with the modified Clauss method. Their data demonstrate argatroban at increasing plasma (molar) concentrations caused a significant decline in plasma fibrinogen levels when using the Clauss method using two different machines/assays. Critical to hemostasis, fibrinogen levels must be maintained post-cardiac surgery [7].

Zhang et al. conducted a study where they added increasing argatroban concentrations (0.3-2 µg/ml) to normal pooled plasma from 20 subjects. The samples were analyzed by multiple fibrinogen assays with three different methods, including the Clauss method using ACL-TOP (Beckman Coulter, Brea, CA), amongst others. Other methods included PT-derived and N Antiserum to Human Fibrinogen reagent (Dade Behring Inc., Deerfield, IL). Their results showed a significant reduction of fibrinogen using the Hemosil Fibrinogen-C XL reagent (Instrumentation Laboratory, Bedford, MA), via the Clauss method on ACL-TOP (P<0.01) based on aPTT ratios. This included a significant reduction of fibrinogen values of up to 96% compared to baseline (P<0.01) [4].

The effects of argatroban on fibrinogen assays differ greatly, thereby leading to possible misrepresented fibrinogen readings. Upon further investigation, we learned that in our institution the Clauss method is used to measure fibrinogen and this explains the rapid drop in fibrinogen in our case. It is important for health care providers to recognize this possibility, as this can lead to inappropriately looking for possible hemorrhagic states like DIC, and thus delay much needed anticoagulation therapy. In the setting of argatroban use and significantly reduced fibrinogen levels, health care providers should check which assay and method their institute/lab use to detect fibrinogen levels. 

## Conclusions

Any change in a lab value that is not clinically explained should always alert the provider to check for the possibility of in vitro lab error. Argatroban may falsely reduce measured fibrinogen levels in vitro, caused by the Clauss method. Though clinically not significant, argatroban should be stopped in such conditions, in order to decrease the chance of mismanagement.
